# A Case Report of B-cell Lymphoblastic Leukemia/Lymphoma Presenting as Isolated Torticollis in a 2-year-old Female

**DOI:** 10.5811/cpcem.2020.8.48524

**Published:** 2020-10-19

**Authors:** Marina Boushra

**Affiliations:** East Carolina University School of Medicine, Department of Emergency Medicine, Greenville, North Carolina. Vidant Medical Center, Department of Emergency Medicine, Greenville, North Carolina. Vidant Beaufort Hospital, Department of Emergency Medicine, Washington, North Carolina

**Keywords:** atraumatic torticollis, malignancy

## Abstract

**Introduction:**

Malignancy is a rare cause of acquired torticollis in children, and spinal cord involvement from hematolymphoid malignancies is similarly unusual. Neurologic abnormalities may not be present on initial evaluation, and delayed diagnosis and treatment is associated with increased risk of permanent paralysis.

**Case Report:**

The author describes a case of isolated torticollis in a 2-year-old evaluated multiple times in the emergency department (ED) and outpatient settings. For her first three presentations, the patient had no associated neurologic abnormalities. She was discharged with return precautions and a presumptive diagnosis of viral infection/lymphadenitis. She later developed weakness of her left arm and was diagnosed with a B-cell lymphoblastic leukemia/lymphoma causing spinal cord compression.

**Conclusion:**

This case highlights the importance of continued comprehensive and meticulous physical examination in patients with repeat ED visits, as well as the value of detailed discharge instructions in mitigating diagnostic delays in these patients.

## INTRODUCTION

Evaluation of pediatric neck complaints in the emergency department (ED) is challenging, as the differential is broad and a complete history is often difficult to obtain in pre-verbal patients. History and physical examination are sufficient for diagnosis in most patients. However, when additional evaluation would be helpful, it is often hindered by concerns over radiation exposure in pediatric patients, limited availability of magnetic resonance imaging (MRI) capabilities, and the need for sedation to obtain high-quality images in some pediatric patients.^1^,^2^

Acquired torticollis is a common complaint in pediatric patients and is caused by trauma or infection in the vast majority of cases.^3^ Malignancy is a rare cause of torticollis in children, and hematolymphoid malignancies can rarely present with spinal cord involvement.^4^,^5^ Early in the clinical course, these patients may not have associated neurologic or laboratory abnormalities.^6^,^7^ Here the author presents a case of a patient with painless atraumatic torticollis without associated neurologic abnormalities evaluated in the ED who was later diagnosed with B-cell lymphoblastic leukemia/lymphoma with cervical cord compression.

## CASE REPORT

A previously healthy 2-year-old female presented to the ED with her mother for neck pain and limited neck rotation that had started that day. At the time of her first evaluation, she had normal vital signs, normal movement of her neck, no lymphadenopathy, a normal neurologic examination, and rhinorrhea. The mother expressed concern that her symptoms were occurring secondary to an impact to her neck when she struck a table the prior day. Both traumatic and viral causes of torticollis were considered in this patient. Due to the presence of rhinorrhea and the lack of neurologic or traumatic findings, the patient was diagnosed with a presumptive viral infection and discharged with instructions for supportive care and routine follow-up.

The patient re-presented to the ED with her father two days later with complaint of inability to rotate her head to the right. She was receiving acetaminophen at home without improvement in her symptoms. Her behavior and appetite were unchanged from her baseline. In the ED, her vital signs were again normal and her exam was notable for head deviation to the left with increased tone of the left sternocleidomastoid and multiple enlarged, non-tender, matted lymph nodes in the left anterior cervical chain. A review of systems was notably negative for any recent fevers, changes in appetite or weight, rashes, or bruising. Her neurologic examination was normal and the remainder of her examination, including auscultation over the carotids and a pharyngeal examination, was unremarkable. She showed some improvement with ibuprofen and diazepam and was discharged with a presumptive diagnosis of viral vs traumatic torticollis.

The patient then presented to her primary care provider four days later with persistent inability to rotate her neck. Her exam was unchanged at that time and she was discharged with a diagnosis of viral lymphadenitis and instructions for continued supportive care and a follow-up appointment four days later. At her follow-up appointment her symptoms persisted, and because her oral intake had decreased she was referred for admission. Her admission labs, including a complete blood count with differential, were normal. A computed tomography (CT) of the soft tissue of the neck with contrast was obtained and initially read as unremarkable. She remained hospitalized for four days for pain control and physical therapy without improvement in the range of motion of her neck. On the fourth day of her hospitalization, she developed weakness in her left arm and increasing lethargy. This prompted re-examination of her initial CT, where a possible spinal cord tumor was noted (Image, panel A). Follow-up MRI showed an extramedullary tumor extending from the level of the second cervical vertebra (C2) to the seventh cervical vertebra (C7) with associated cord edema (Image, panel B).

CPC-EM Capsule

What do we already know about this clinical entity?

*The vast majority of acquired pediatric torticollis presentations occur secondary to trauma or viral infection*.

What makes this presentation of disease reportable?

*This is a case of painless, atraumatic torticollis without neurological abnormalities ultimately diagnosed as B-cell lymphoma with cervical cord compression*.

What is the major learning point?

*Torticollis may precede the presence of neurological symptoms in pediatric cervical spine malignancies*.

How might this improve emergency medicine practice?

*This case illustrates the importance of repeat neurologic examinations with repeated presentations and detailed return precautions in pediatric torticollis*.

Given her declining neurologic status, she was given dexamethasone for the cord edema. A cervical mass biopsy and bone marrow biopsy done 72 hours later were both inconclusive. Repeat MRI showed complete resolution of the mass with the steroids. Based on this response to steroids, a presumptive diagnosis of leukemia/lymphoma was made and the patient was discharged on dexamethasone. Four days later, she presented to the pediatric ED again, this time with left facial droop and weakness of the upper and lower extremities on the left side. Examination was notable for swelling of the right wrist and left leg. A lytic lesion was noted on radiograph of the right ulna. An iliac biopsy was again non-diagnostic. On the second day of her hospitalization, she also developed subcutaneous scalp nodules. Biopsies and flow cytometry of the nodules were consistent with B-cell lymphoblastic leukemia/lymphoma. The patient was started on chemotherapy and is currently doing well, with strength in her extremities improving with physical therapy.

## DISCUSSION

The differential of acquired torticollis is broad and includes a variety of benign and eminently life-threatening conditions. The vast majority of cases are caused by self-limiting infections and trauma. Conditions such as retropharyngeal abscesses and atlantoaxial rotary subluxation are uncommon but important considerations that need urgent management. Central nervous system malignancies account for only an estimated 2% of pediatric cases of acquired torticollis.^3^ However, one retrospective review of pediatric patients under 13 years of age with tumors of the posterior fossa or cervical spine noted acquired, atraumatic torticollis as the presenting symptom in 22% of the cases.^6^ In the sub-group including only cervical spine malignancies, like the reported patient, 10% presented with isolated torticollis.^6^ In all cases in this study, torticollis preceded the onset of neurologic symptoms, and an average of 9.5 weeks passed before neurologic manifestations developed and led to a diagnosis of malignancy.^6^ The mechanism of torticollis in cervical cord tumors is poorly understood but is theorized to be secondary to compression of the spinal accessory nerve (cranial nerve XI) by the mass.^4^

Neurologic examination is a critical part of the evaluation of any patient with neck complaints. Any abnormality in neurologic examination is a “red flag symptom” that should prompt additional evaluation, typically with imaging of the neck. However, studies have shown that torticollis can precede the onset of neurologic findings by days or weeks in malignancies of the cervical spine and posterior fossa. Concern for the radiation exposure associated with CT imaging of the spine in children precludes routine imaging of these patients.^1^,^2^ MRI is costly, not typically feasible in the ED setting, and may require sedation in young children.^8^–^10^

While clinical decision tools such as the Pediatric Emergency Care Applied Research Network criteria and the Pediatric Appendicitis Score have aided in decreasing unnecessary imaging in certain pediatric conditions, there are no guidelines or risk-stratification tools to aid emergency physicians in determining the need for additional evaluation in patients presenting with atraumatic torticollis without neurologic deficits.^10^ Given the rarity of this diagnosis, the paucity of physical examination findings early in the course, and the risks of unnecessary imaging in pediatric patients, some diagnostic delay may, unfortunately, be inevitable. However, such cases serve as a reminder of the importance of considering malignancy in patients presenting with atraumatic torticollis, repeating thorough neurologic examinations with repeated ED presentations, and providing detailed return instructions to parents.

## CONCLUSION

Acquired torticollis is a common pediatric ED presentation that is usually caused by infection or trauma. Neurologic examination is an essential component of the evaluation of any patient presenting with neck complaints. However, up to 10% of patients with cervical cord malignancy can present with sudden-onset, acquired torticollis without evidence of neurological abnormalities. It is understandably difficult to risk-stratify these patients for imaging given the rarity of this diagnosis and the paucity of physical examination findings. This case of a hematolymphoid malignancy with central neurologic involvement highlights the importance of continued comprehensive and meticulous physical examination in patients with repeat ED visits, as well as the value of detailed discharge instructions. While an uncommon cause and presentation, malignancy of the central nervous system should be considered as a cause of sudden-onset torticollis in pediatric patients and additional evaluation, including MRI, should be considered in patients with sudden onset of symptoms or those with a protracted course.

## Figures and Tables

**Image:**
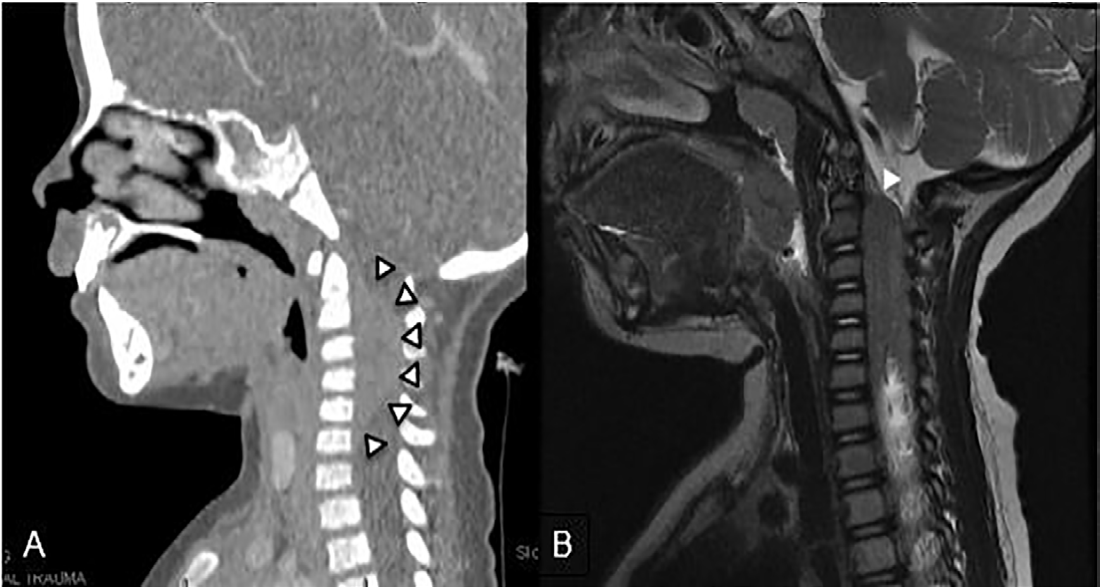
(A) Computed tomography with contrast of neck soft tissue, initially read as containing no pathology. Note the subtle hyperlucent lesion extending from the level of the second cervical vertebra (C2) to the seventh cervical vertebra (C7) (black-edged white arrows), corresponding to the hypodense region on the magnetic resonance imaging (MRI) (B). MRI with contrast of the cervical spine showing a heterogeneously enhancing mass extending from C2 to C7 with edema and cord flattening (white arrow).
